# Case Report: ^18^F-PSMA-1007 PET/CT Avid Solitary Penile Metastasis of Castration-Resistant Prostate Cancer With a PSA of 0.072 ng/ml

**DOI:** 10.3389/fonc.2022.881896

**Published:** 2022-04-20

**Authors:** Yongliang Li, Yanmei Li, Siying Dong, Jian Chen, Pengfei Yang, Juan Li

**Affiliations:** ^1^Department of Nuclear Medicine, General Hospital of Ningxia Medical University, Yinchuan, China; ^2^School of Clinical Medicine, Ningxia Medical University, Yinchuan, China

**Keywords:** penile metastasis, ^18^F-PSMA-1007 PET/CT, prostate cancer, PSA, castration resistance

## Abstract

Penile metastasis of prostate cancer is rare, with a poor prognosis, and only a limited number of relevant cases have been reported so far. With the application of ^18^F-PSMA-1007 PET/CT, the biochemical recurrence of prostate cancer can be detected at an early stage for providing important evidence, facilitating clinical decision-making. Here, we have reported a case of solitary penile metastatic recurrence in the context of mild PSA progression (PSA: 0.072 ng/ml). This case highlights the preferable sensitivity of ^18^F-PSMA-1007 PET/CT imaging in prostate cancer.

## Background

Secondary penile tumors are rare and have a poor prognosis, with a mortality rate of 80% within 6 months, 28% of which is accounted for by prostate cancer (1–3). Previous literature has reported that penile metastasis occurs mainly secondary to primary prostate cancer without any medical treatment (4, 5) or occurs during an androgen-deprivation therapy (ADT) without surgery (6, 7). However, the present case represents a biochemical recurrence of prostate cancer in the penis during ADT after radical prostatectomy with a PSA of 0.072 ng/ml, which has not been reported in the literature so far.

## Case Presentation

**Figure 1:**
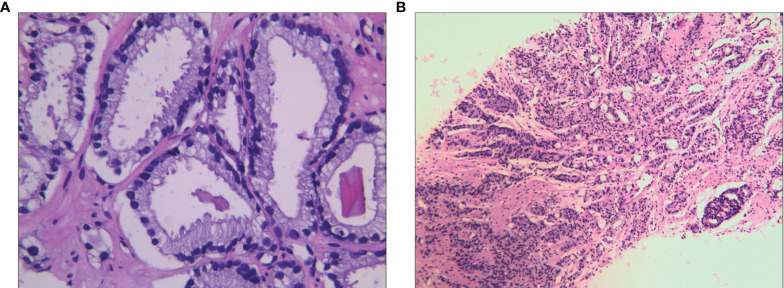
Adenocarcinoma of the prostate with a Gleason score of 3 + 4 = 7 **(A, B)**.

**Figure 2:**
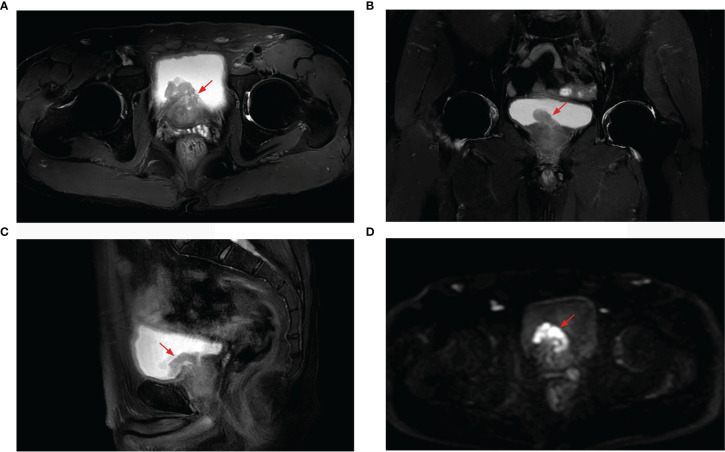
Prostate MRI revealing prostate cancer with bladder invasion, a marked hyperintensity on T2-weighted imaging [**(A)** axial, **(B)** coronal, **(C)** sagittal], and hypointensity on diffusion-weighted imaging **(D)**.

**Figure 3:**
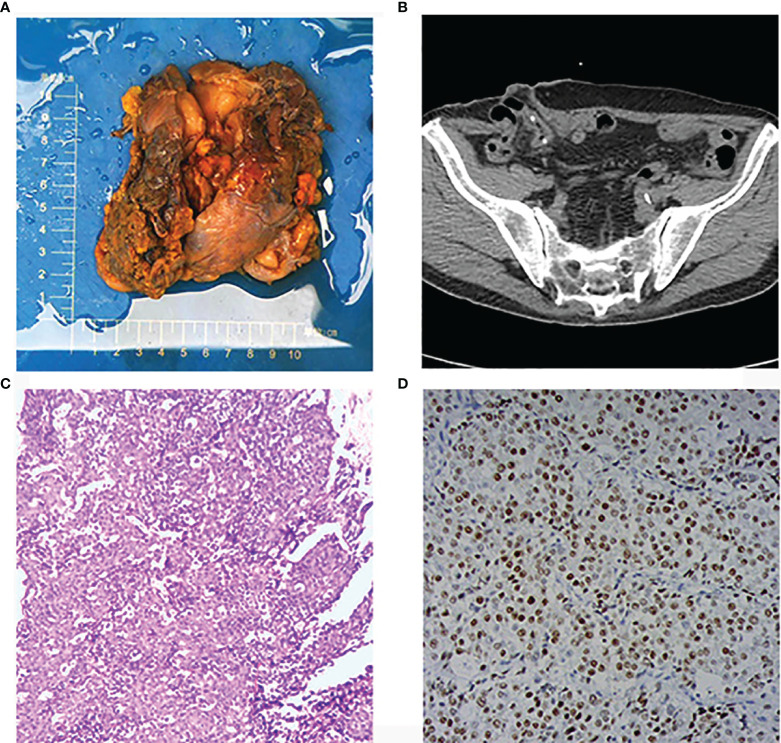
**(A)** Gross specimen of the prostate with a small bladder tissue. **(B)** Ileocystoplasty was performed and a new urinary excretory system was reconstructed. **(C)** H&E staining of bladder invasion lesions showing poorly differentiated prostatic adenocarcinoma with a Gleason score of 5 + 5 = 10. **(D)** Immunohistochemical staining result, NKX3.1(+).

**Figure 4:**
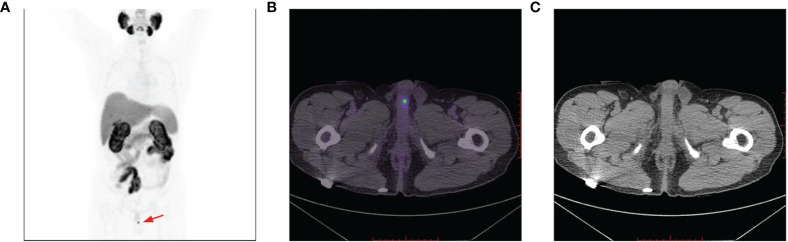
A ^18^F-PSMA-1007 PET/CT avid solitary penile lesion with a SUV_max_ of 6.4 **(A, B)**; no morphological abnormalities of the penis detected on CT imaging **(C)**.

**Figure 5:**
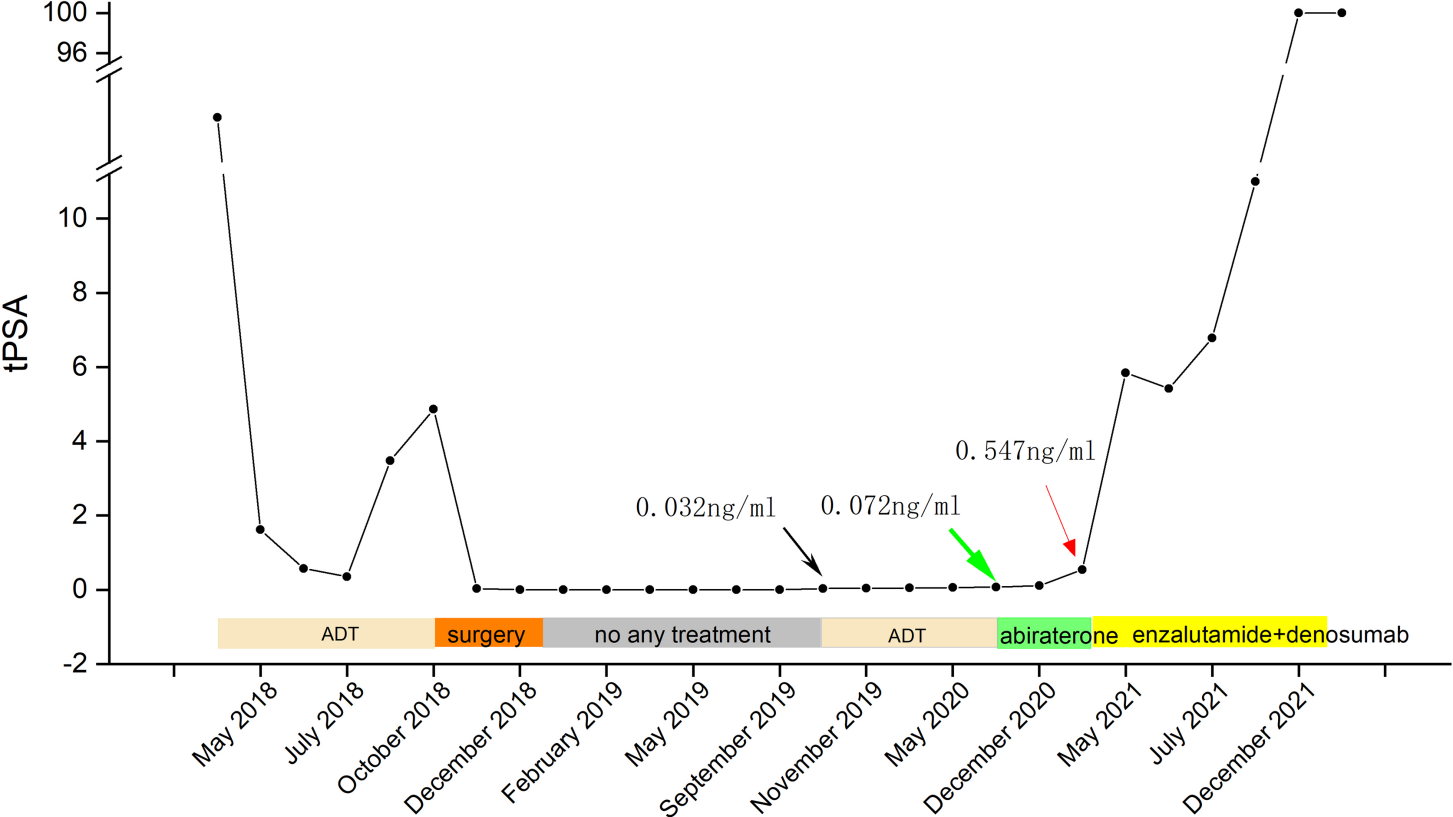
Timeline of the PSA level and treatment (green arrow—a positive result of ^18^F-PSMA-1007 PET/CT imaging with a PSA of 0.072 ng/ml; black arrow—an elevated PSA of 0.032 ng/ml; red arrow—an elevated PSA of 6.79 ng/ml).

**Figure 6:**
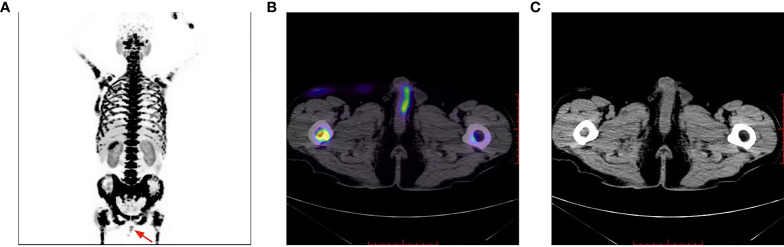
Penile lesion was enlarged with a higher SUV_max_ of 7.4 **(B)**; meanwhile, systemic bone metastasis was certifified **(A)**. No morphological abnormalities of the penis detected on CT imaging **(C)**.

A 60-year-old man visited the hospital with the complaint of intermittent urethralgia and urine arrest. The preliminary screening revealed an elevated PSA of 40.688 ng/ml. Accordingly, prostate cancer was suspected and needle biopsy was performed, followed by confirmation of the adenocarcinoma of the prostate with a Gleason score of 3 + 4 = 7 (**Figure 1**). Considering the patient’s unwillingness to undergo surgery and external beam radiotherapy (EBRT), the ADT was adopted with bicalutamide and leuprorelin. PSA decreased significantly at the beginning of treatment, but increased gradually as the treatment progressed, to reach 4.86 ng/ml in October 2018. Prostate magnetic resonance imaging (MRI) confirmed prostate cancer with bladder invasion (**Figure 2**). The patient’s condition was evaluated comprehensively, and radical prostatectomy and cystectomy were conducted. Adenocarcinoma of prostate cancer combined with nerve and vascular invasion was confirmed with an elevated Gleason score of 5 + 5 = 10, and tumor invasions of the bladder neck, bladder mucosa, and submucosal muscularis were also observed. For the reconstruction of the urinary excretory system, ileocystoplasty was performed (**Figure 3**). A stable serum PSA level (PSA ≤ 0.03 ng/ml) was maintained to reveal a favorable prognosis within a year of surgery. However, a slightly elevated PSA level was noted with a value of 0.035 ng/ml in October 2019, for which ADT with bicalutamide and leuprorelin was undertaken. Biochemical recurrence was suspected, and PET/CT was performed after injection with 11.95 mCi (442.1 MBq) ^18^F-PSMA-1007 in August 2020 on the recommendation of the patient’s physician with a PSA of 0.072 ng/ml. Surprisingly, no other PSMA-avid foci were located in the prostatic bed and the pelvis, except for an intense uptake in the corpus cavernosum with a SUV_max_ of 6.4 (**Figure 4**). Considering the poor efficacy of ADT, abiraterone was applied; meanwhile, the usage of bicalutamide and leuprorelin was discontinued. Subsequently, an elevated PSA level was recorded (0.547 ng/ml) in March 2021 and in July 2021 (6.79 ng/ml) (**Figure 5**). Enzalutamide combined with denosumab was applied, but abiraterone was discontinued. After several months, the patient showed a marked increase in PSA value (>100 ng/ml), combined with penile bleeding. ^18^F-PSMA-1007 PET/CT revealed that the uptake of penile lesions was significantly higher (SUV_max_ 7.4) and that the range of lesions was enlarged than earlier; meanwhile, systemic bone metastasis was detected (**Figure 6**). Considering the poor prognosis, the patient refused to undergo penectomy, and hence palliative chemotherapy was adopted.

## Discussion and Conclusion

Penile metastasis of prostate cancer is rare (8, 9), with only a few PSMA-targeted imaging presented in the literature (7, 10, 11), none of which underwent radical prostatectomy, and only ADT and external beam radiotherapy were performed in these cases. Castration resistance develops over time and the tumor can recur. However, in the present case, a solitary biochemical recurrence of prostate cancer occurred in the penis during ADT after radical prostatectomy, while no recurrence was noted in the prostate bed and pelvis. The patient progressed to systemic bone metastasis despite undertaking a full course of androgen deprivation, which indicated castration resistance (12). Because the patient underwent ileocystoplasty surgery, urine was not excreted through the urethra, and hence the possibility of false-positive results contributed by penile radioactive retention can be ruled out. Unfortunately, due to the extensive metastasis and poor prognosis, the patient did not undergo further biopsy and penectomy. The case of biochemical recurrence after prostatectomy with a relatively low serum PSA level of 0.072 ng/ml may have been affected by a sequential ADT, which possibly reduced the activity of a recurrent lesion. However, owing to the high sensitivity of ^18^F-PSMA-1007 PET/CT imaging, biochemical recurrence lesions could be detected at an early stage. As Giesel et al. (13) reported, ^18^F-PSMA-1007 PET/CT detected biochemical recurrence with a PSA level of 0.08 ng/ml, which, in turn, provided important evidence for clinical decision-making.

## Data Availability Statement

The raw data supporting the conclusions of this article will be made available by the authors, without undue reservation.

## Ethics Statement

The studies involving human participants were reviewed and approved by the Scientific Research Ethics Committee of Ningxia Medical University General Hospital. The patients/participants provided their written informed consent to participate in this study. Written informed consent was obtained from the individual(s) for the publication of any potentially identifiable images or data included in this article.

## Author Contributions

All authors listed have made a substantial, direct, and intellectual contribution to the work and approved it for publication.

## Funding

This study was supported by Natural Science Foundation of Ningxia Hui Autonomous Region (No. 2021AAC03385).

## Conflict of Interest

The authors declare that the research was conducted in the absence of any commercial or financial relationships that could be construed as a potential conflict of interest.

## Publisher’s Note

All claims expressed in this article are solely those of the authors and do not necessarily represent those of their affiliated organizations, or those of the publisher, the editors and the reviewers. Any product that may be evaluated in this article, or claim that may be made by its manufacturer, is not guaranteed or endorsed by the publisher.
